# Determinants of visual acuity outcomes in eyes with neovascular AMD treated with anti-VEGF agents: an instrumental variable analysis of the AURA study

**DOI:** 10.1038/eye.2016.90

**Published:** 2016-05-20

**Authors:** F G Holz, R Tadayoni, S Beatty, A R Berger, M G Cereda, P Hykin, G Staurenghi, K Wittrup-Jensen, J Nilsson, K Kim, S Sivaprasad

**Affiliations:** 1 Department of Ophthalmology, University of Bonn, Bonn, Germany; 2 Department of Ophthalmology, Hôpital Lariboisière, Paris, France; 3 Department of Ophthalmology, Institute of Eye Surgery, Waterford, Ireland; 4 Department of Ophthalmology and Vision Sciences, University of Toronto, and St Michael's Hospital, Toronto, Ontario, Canada; 5 Department of Biomedical and Clinical Science Luigi Sacco, Luigi Sacco Hospital, University of Milan, Milan, Italy; 6 NIHR Biomedical Centre for Research in Ophthalmology, Moorfields Eye Hospital, London, UK; 7 Bayer Pharmaceuticals, Berlin, Germany; 8 Mapi Group, Real World Strategy and Analysis, Stockholm, Sweden; 9 Health Economics, AstraZeneca Nordic-Baltic, Södertälje, Sweden; 10 Department of Ophthalmology, King's College Hospital, London, UK

## Abstract

**Purpose:**

To identify the strongest variable(s) linked with the number of ranibizumab injections and outcomes in AURA, and to identify ways to improve outcomes using this association.

**Methods:**

AURA was a large observational study that monitored visual acuity over a 2-year period in patients with neovascular age-related macular degeneration (AMD) who received ranibizumab injections. Baseline characteristics, resource use, and outcomes were analyzed using an instrumental variable approach and regression analysis.

**Results:**

Data were analyzed from 2227 patients enrolled in AURA. Optical coherence tomography (OCT) and ophthalmoscopy were the most common diagnostic tests used, and this combination was the strongest instrumental variable. Use of OCT and ophthalmoscopy affected the number of injections given and resulted in an increase in visual acuity gains from baseline of 17.6 letters in year 1 and 2.5 letters in year 2. Regression models using the instrumental variable (OCT and ophthalmoscopy combined) showed that ≥5.1 (95% CI: 3.3–11.4) ranibizumab injections were needed to maintain visual acuity from baseline to year 1 and ≥8.3 (95% CI: 5.3–18.8) injections were needed to maintain visual acuity from year 1 to year 2. To gain ≥15 letters, ≥7.9 (95% CI: 5.1–17.5) ranibizumab injections would be needed in year 1 and ≥16.1 (95% CI: 10.3–36.4) injections would be needed over 2 years.

**Conclusions:**

These findings highlight the role that regular monitoring plays in guiding neovascular AMD therapy and they showed that the number of ranibizumab injections needed to maintain visual acuity is higher than that administered in AURA.

## Introduction

Anti-vascular endothelial growth factor (VEGF) agents have become an important treatment option for neovascular age-related macular degeneration (AMD) since their introduction over a decade ago. These agents inhibit VEGF, a key factor in the development of underlying cell proliferation and neovascularization.^1^ Improvements in visual and anatomical outcomes following monthly injections of the anti-VEGF antibody fragment ranibizumab have been demonstrated in two key studies in neovascular AMD.^2, 3^ However, delivery of monthly dosing in clinical practice is challenging, and alternative dosing regimens of intravitreal ranibizumab are often used, including as-needed, quarterly, or treat-and-extend, although outcomes can be more variable.^4–10^ Not surprisingly, observational studies have shown that ranibizumab may be underused in routine practices, resulting in poor long-term outcomes.^11–13^

AURA (a retrospective noninterventional study to assess the effectiveness of existing Anti-vascUlar endothelial growth factor treatment Regimens in patients with wet Age-related macular degeneration) monitored 2-year outcomes in patients with neovascular AMD who started treatment with ranibizumab between January 2009 and August 2009.^11^ This observational study showed that visual acuity gains were not maintained over 2 years, and the mean number of injections was low. Initially, we performed logistical regression analysis of AURA, and found that inadequate monitoring, injections, and use of diagnostic tools compromised treatment outcomes.^12^

To further determine the relationship between ranibizumab injections and outcome in a nonrandomized setting, such as AURA, we also performed an instrumental variable analysis of this data set. This approach is validated and well established, and is used to identify causal relationships in a noncontrolled situation that is often subject to bias and confounding from both measured and nonmeasured variables. Notably, the instrumental variable method has the potential to adjust for these confounders, making it ideal for evaluating observational data.^14, 15, 16, 17, 18, 19, 20^ In this case, we identified the (instrumental) variable from the AURA data set (ie, baseline characteristics and resource use) that had the strongest causal link with number of ranibizumab injections and outcome (mean change in visual acuity (letters) at year 1 and year 2). Any confounding between the instrumental variable identified and the outcome was tested through application of the Wald estimator (which statistically tests the true value of the parameter based on the sample estimate). To test whether the selection was random, the F-statistic was also applied; this test identifies the level of bias in the sample. The outcomes from the instrumental variable analysis of the AURA data are reported in this paper.

## Materials and methods

AURA was a retrospective, observational, multicenter study conducted in eight countries (Canada, France, Germany, Ireland, Italy, the Netherlands, the United Kingdom, and Venezuela). The design has been described in detail elsewhere.^11, 12^ In brief, its primary aim was to evaluate changes in visual acuity in patients who started ranibizumab therapy between January 2009 and August 2009. The overall (exposed) population consisted of those who received at least one dose of anti-VEGF treatment, whereas the effectiveness analysis set consisted of patients who additionally had at least one post-baseline assessment of visual acuity for the treated eye. The first-year and second-year completer analysis sets included those in the effectiveness analysis set for whom follow-up data for ≥1 and ≥2 years after first injection, respectively, were documented. Because of the exploratory nature of the study, the statistical analysis was descriptive. To account for missing data, mean change in visual acuity was assessed using a last-observation-carried-forward analysis.

### Objectives

The aim of this paper is to apply an instrumental variable analysis to the AURA data set in order to identify the strongest instrumental variable(s) linked with the number of ranibizumab injections and outcomes (mean change in visual acuity (letters) at year 1 and year 2), and to identify ways to improve visual acuity outcomes using this association.

### Analyses

The analyses were performed using StataCorp LP 2007 (Stata Statistical Software: Release 10, College Station, TX, USA). The AURA data set and definitions used in this paper have been described elsewhere.^12^ The baseline characteristics, health insurance, reimbursement, and resource utilization (in year 1 and over 2 years) by country are summarized in this paper. Resource utilization included number of ophthalmoscopies, optical coherence tomography (OCT) images, fluorescein angiographies, and indocyanine green angiographies. Monitoring (diagnostic assessments only) and clinic (scheduled treatment) visits were also reported. Any differences between country data were analyzed using either ANOVA (for continuous variables that were normally distributed) or *χ*^2^ test (for categorical variables).

Given the volume of variables included in AURA, Pearson's correlation coefficient (95% confidence interval (CI)) was initially used to test the strength of correlation between variables and number of ranibizumab injections. The instrumental variable analysis (summarized in Supplementary Figure 1) that was applied to the candidate variables identified has been described in numerous publications.^14–20^ A standard approach was used; that is, we applied a series of linear equations (listed below). Using the F-statistic (to test for random assignment), the variable with the strongest correlation to the number of ranibizumab injections was selected as the instrumental variable. The Wald estimator was also used (to evaluate the validity of the instrumental variable in relation to the number of injections and outcomes). The instrumental variable approach was then applied to test the relationship between the number of ranibizumab injections and outcomes using regression analysis. The analysis was developed using known assumptions (described below).

#### Testing random assignment

The relationship between the instrumental variable (ie, a variable with high correlation with the exposure but low correlation with the outcome) and exposure (the number of ranibizumab injections) should not be confounded by other variables. The F-statistic of the regression (equation (1)) estimates the magnitude of bias in terms of confounding by other variables.^17^ In equation (1) ρ̂_Z,X_^2^ refers to the square of the estimated correlation coefficient between the instrumental variable (*Z*; 1 × *n*) and the exposure (*X*; 1 × *n*) and *n* is the sample size. An F-value not far from 1 indicates a high risk of small sample bias, whereas a value of 10 seems to be sufficient for the bias to be negligible:^18^


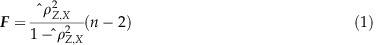


#### Evaluating the validity of the instrumental variable in relation to the number of injections and outcomes

The Wald estimator was used to evaluate the overall validity of the instrumental variable.^14, 17^ The Wald estimator statistically tests the true value of the parameter based on the sample estimate. In the case of a dichotomous instrument and exposure, the Wald estimator is given by equation (2). The numerator of this estimator is an intention-to-treat estimator (ie, the effect of the instrument on the outcome measured as a risk difference). The denominator is the difference in treatment rates between levels of the instruments (eg, treatment arms of the randomized controlled trial) and is a measure of compliance. As the noncompliance increases, the denominator shrinks and the instrumental variable estimator increases relative to the intention-to-treat estimator.


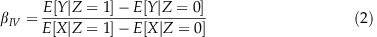


where *X*=exposure, *Y*=outcome, and *Z*=instrumental variable.

Regression models using the validated instrumental variable were developed; this method has been applied in other studies.^21, 22^ In brief, a two-stage least-squares approach was used to calculate the instrumental variable estimates. In the first stage, each explanatory variable that is an endogenous covariate in the equation of interest is regressed on all of the exogenous variables in the model, including both exogenous covariates in the equation of interest and the excluded instrumental variable. The predicted values from these regressions are obtained using the following equations (equations (3) and (4)), where *Z* (column vector with *n* objects) and *X* (matrix with all covariates for *n* objects) are the instrumental variable and the covariates, respectively; δ̂ includes the estimated regression coefficients, *T* is the letter indicating the transpose of the vector, and *X̂* contains the predicted *X* values.

Stage 1: regress each column of *X* on *Z*:


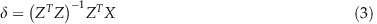


and save the predicted values:


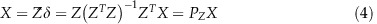


In the second stage, the regression of interest is estimated as usual, except that in this stage each endogenous covariate is replaced with the predicted values from the first stage based on the following equations (equations (5) and (6)), where *Y* is the outcome, *X̂* is the predicted *X*, *β* are the regression coefficients, and *ϕ* is the error.

Stage 2: regress *Y* on the predicted values from the first stage:


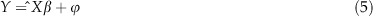


which gives the two-stage least-square estimator *β*_2*SLS*_,where *P*_*z*_ = *Z*(*Z*^*T*^*Z*)^−1^*Z*^*T*^:


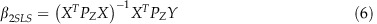


## Results

### Participants

In the AURA study, 2227 patients were included in the effectiveness analysis set, and 1695 patients completed year 1 and 1184 patients completed year 2. Baseline characteristics for the effectiveness analysis set are shown in Table 1a. There were statistically significant differences between countries in potential confounding variables such as mean age at start of therapy that ranged from 73 years (Ireland and Venezuela) to 80 years (Canada); health insurance (public health insurance ranged from 99% in the Netherlands to 2% in Ireland); reimbursement (national standard was achieved by 96% in the Netherlands and Ireland, and by 15% in Venezuela); and mean baseline visual acuity score (VAS) that ranged from 47 letters in Canada to 66 letters in Italy.

Resource utilization by country is summarized in Table 1b. There were statistically significant differences between countries in the number of ranibizumab injections, diagnostic tests, and visits. In year 1, the mean number of ranibizumab injections ranged from 3.4 (Venezuela) to 7.4 (Ireland), and clinic visits ranged from 3.8 (Venezuela) to 8.1 (Ireland). The mean number of OCT examinations ranged from 2.5 (Venezuela) to 9.3 (United Kingdom) in year 1, and from 5.2 (Venezuela) to 17.5 (United Kingdom) over 2 years. Spectral-domain OCT was used in 64.7% of first-year completers and 63.3% of second-year completers. Time domain OCT was used in 33.1% of first-year completers and 34.2% of second-year completers.

### Identification of candidate instrumental variables

The Pearson's correlation coefficient was used as the primary criterion to test the correlation between the number of ranibizumab injections and candidate instrumental variables (Table 2). Treatment duration was the variable most strongly correlated with number of injections, with a correlation coefficient of 0.6; however, as treatment duration can be related to the outcome, it was not chosen as the instrumental variable. The number of OCT and ophthalmoscopy examinations performed was also correlated with the number of injections; the correlation coefficients were 0.4 and 0.3, respectively.

According to clinical guidelines and published literature, OCT is recommended for screening the macula before performing more invasive imaging such as fluorescein angiography.^23, 24, 25^ OCT alone may be able to provide sufficient information to facilitate follow-up decisions, and is considered the essential diagnostic and monitoring tool in the treatment of neovascular AMD. The number of OCTs performed is, therefore, the most clinically relevant instrumental variable. At the time of this study, OCT was the most frequently used diagnostic and monitoring tool in France, the United Kingdom, Ireland, Italy, the Netherlands, and Venezuela, and ophthalmoscopy was the dominant diagnostic and monitoring tool in Germany and Canada (Table 1b). Therefore, a combination of OCT and ophthalmoscopy was used as the most appropriate instrumental variable across countries, resulting in a correlation coefficient of 0.4 (Table 2).

The suitability of using OCT and ophthalmoscopy as an instrumental variable was further evaluated using the Wald estimator. As the Wald estimator commonly uses a binominal variable for the instrumental variable in the equation (equation (2)), the number of OCT and ophthalmoscopy examinations was dichotomously grouped by the median number of OCT and ophthalmoscopy examinations performed at year 1, and over the 2-year period. The variables 0 and 1 were used to represent <8 and ≥8 OCT and ophthalmoscopy examinations in year 1, and <19 and ≥19 OCT and ophthalmoscopy examinations over the 2-year period. In this equation, the exposure was defined as the number of ranibizumab injections and the outcome was the number of letters gained. This analysis showed that a higher number of OCT and ophthalmoscopy examinations affected the number of injections and resulted in an increase from baseline of 17.6 letters in year 1 and 2.5 letters in year 2. When the Wald estimator was used in relation to the number of ranibizumab injections and letters gained from baseline, the effect of the number of OCT and ophthalmoscopy examinations was 13.9 letters in year 1 and 2.4 letters in year 2. Given that the clinical significance for letters gained in visual acuity is ±15 letters, the Wald estimates suggest that the effect of the instrumental variable on letters gained in visual acuity is significant in year 1 but not in year 2.

The regression models using the instrumental variable (OCT and ophthalmoscopy combined) are presented in Table 3a and b. For both analyses, the values for the F-statistics were >10 in the first-stage model, indicating that the regression model was stable. The regression model also indicates that the average patient aged 76.9 years with a baseline VAS of 56.9 letters would lose 27.8 letters from baseline to year 1 without any ranibizumab injections (ie, –3.76 + (–0.279 × 56.9) + (–0.106 × 76.9)). At least 5.1 (95% CI: 3.3–11.4) ranibizumab injections would be needed to maintain visual acuity from baseline to year 1. Similarly, the average patient aged 77.0 years with a baseline VAS of 57.2 letters would lose 16.0 letters from year 1 to year 2 without any ranibizumab injections (ie, 19.953 + (–0.391 × 57.2) + (–0.177 × 77)). At least 8.3 (95% CI: 5.3–18.8) ranibizumab injections would be needed to maintain visual acuity from year 1 to year 2. To gain ≥15 letters, ≥7.9 (95% CI: 5.1–17.5) ranibizumab injections would be needed in year 1 and ≥16.1 (95% CI: 10.3–36.4) ranibizumab injections would be needed over 2 years.

## Discussion

AURA monitored the routine use of ranibizumab injections for the treatment of neovascular AMD in clinical practices from eight countries. Overall, patients gained +2.4 letters in year 1 that declined to +0.6 letters in year 2. The mean number of injections was low, decreasing from 5.0 in year 1 to 2.2 in year 2. The mean number of OCTs performed also declined from 4.5 in year 1 to 3.2 in year 2.^11^ These findings indicate that, at the time the AURA study was conducted, outcomes achieved in clinical studies were not translated into clinical practice. In the Comparison of Age-Related Macular Degeneration Treatments Trials (CATT), patients treated with ranibizumab on an as-needed basis maintained gains in visual acuity (+6.7 letters at year 2) and received a mean of 12.6 injections over the 2-year period.^9^ However, it should be noted that CATT was a clinical study with strict inclusion criteria and mandated monthly follow-up; OCT imaging was performed at each visit, and the study protocol described the retreatment criteria. AURA was an observational, retrospective study with no limit to visual acuity and other parameters at baseline.

The current analysis used an instrumental variable to explore potential confounding parameters in this association, and identified that the number of OCT and ophthalmoscopy examinations performed was a strong instrumental variable when testing the relationship between number of injections and letters gained given the acceptable results from F-statistics and Wald estimates. By using the instrumental variable, regression analysis showed that the number of injections is significantly associated with letters gained in visual acuity in year 1 and year 2.

Based on the AURA outcomes, this analysis revealed that the average patient would need ≥5.1 ranibizumab injections to maintain VAS from baseline to year 1 and ≥8.3 ranibizumab injections to maintain VAS from year 1 to year 2. For the average patient to gain ≥15 letters at year 1, ≥7.9 ranibizumab injections would be needed. For the average patient to gain ≥15 letters at year 2, ≥16.1 ranibizumab injections would be needed. It should be noted that these results are only appropriate when either OCT or ophthalmoscopy is assessable during the course of treatment for patients with neovascular AMD.

The analysis has a number of strengths and limitations. An instrumental variable is a useful tool to identify causality where patients are not randomized^26^ and providing that the data set is sufficiently large. Results are not reliable when the instrumental variable does not fulfil one of the required assumptions.^18^ The AURA study, however, fulfills these requirements. If the instrumental variable is correlated with the outcome, generalizability of the overall results is weakened as the instrumental variable is supposed to provide ‘randomized' effects. In our analysis, we removed treatment duration to avoid this, and showed that the number of OCT and ophthalmoscopy examinations combined was the most suitable candidate for an instrumental variable, and that the correlation with the number of injections was strong.

An instrumental variable should be neither directly related to the outcome nor indirectly related via pathways through unmeasured variables. OCT and ophthalmoscopy are diagnostic tools and not treatments, and therefore there should be no direct relationship between the number of OCT and ophthalmoscopy examinations performed and letters gained in visual acuity. However, the use of OCT and ophthalmoscopy examinations as a diagnostic tool may indirectly influence treatment decisions and, thus, treatment outcomes. Indirect relation via pathways through unmeasured variables cannot be tested within the AURA data set. Another limitation is that AURA data on OCT and ophthalmoscopy use were collected during 2009, with a 2-year follow-up. The use of diagnostic tools may have changed in recent years; in Germany, OCT, and not ophthalmoscopy, is now the most frequent diagnostic tool. It must also be noted that treat-and-extend approach has recently become a common approach, but this is unlikely to have been used at the time of the study. The data suggest that a low number of examinations with ophthalmoscopy or OCT imaging or both was used, resulting in a low number of injections and a worse visual acuity outcome. This implies that an as-needed approach was being employed at the time. Given that there has been a shift toward treat-and-extend or extended fixed dosing with increased access to OCT, it is possible that the situation may have improved since the results of AURA were published. However, these results are still applicable when an as-needed approach is preferred. There was also a lack of adjustment for any country-specific differences, including further exploration of reimbursement or health insurance on access to resource. Although access to public health insurance or private health insurance (specifically for Ireland and Venezuela) was evident in all countries, there was wide variation in the use of diagnostic tools and injections. There may be a number of reasons for this, including limitations in insurance coverage. In the United Kingdom, public insurance (National Health Service) is free to all permanent residents, but there are still charges associated with eye tests under some circumstances; this may have resulted in some patients recording ‘no insurance' rather than free public insurance. Finally, only the overall baseline VAS was included as a confounding parameter, and this may have excluded any potential (and clinically relevant) association between higher baseline VAS, fewer injections, and less letter gains.

In conclusion, the instrumental variable method supports the association between the number of injections and letters gained in visual acuity, and also the role that regular monitoring plays in guiding therapy. The regression model showed that the number of ranibizumab injections needed to maintain or gain visual acuity is greater than the mean number of injections administered in the AURA study. This likely explains, at least in part, why the initial visual improvements achieved by patients in AURA were not sustained at year 2. These findings will be of benefit to health-care physicians with an interest in improving treatment decisions for patients with neovascular AMD.


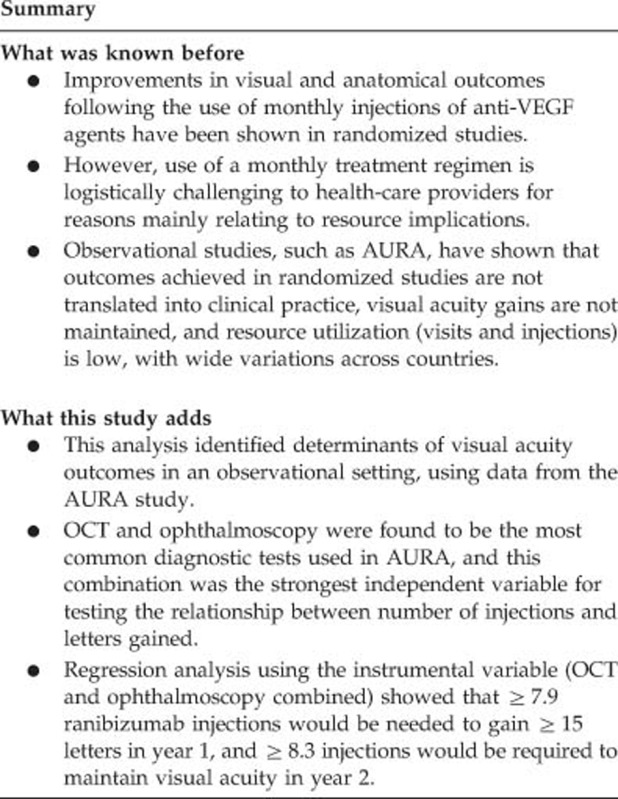


## Figures and Tables

**Table 1:** Baseline characteristics (a) and resource utilization and outcomes over 2 years (b) by country

	*France*	*Germany*	*Canada*	*UK*	*Ireland*	*Italy*	*The Netherlands*	*Venezuela*	*All*	*Difference (*P*-value*^a^)
*(a)*										
*Baseline characteristics (effectiveness analysis set)*										
*n*	398	420	188	410	49	365	350	47	2227	
Age, year	77.5	76.7	79.8	77.7	72.7	75.2	77.2	73.1	76.9	<0.001
Female, %	61	60	61	60	73	58	63	60	61	0.626
Baseline VAS	56.0	52.9	47.2	55.0	64.7	65.5	50.1	48.3	55.4	<0.001
*Lesion type, %*										<0.001
No	2	3	2	0	4	1	2	2	2	
Classic	29	19	10	17	55	27	19	38	22	
Classic and occult	9	16	3	4	4	15	6	6	9	
Occult	33	49	15	25	14	34	33	17	33	
Disciform scar	0	1	2	0	8	0	1	0	1	
Not available	27	12	69	54	14	23	39	36	34	
Prior disease, %	92	95	93	92	88	95	92	98	93	0.140
Concomitant disease, %	87	90	86	95	92	93	86	74	90	<0.001
*Health insurance, %*										<0.001
Public	95	82	93	82	2	89	99	4	86	
Private	2	17	1	1	94	0	1	53	7	
No insurance	0	1	3	15	4	11	0	28	6	
Not available	3	0	3	2	0	0	0	15	1	
*Reimbursement type, %*^b^										<0.001
Individual	2	51	2	1	0	26	3	6	15	
National standards	44	32	92	94	96	73	96	15	69	
Separate contract	0	6	0	0	0	0	0	0	1	
Independently	53	3	1	1	0	1	0	4	10	
Patient	0	0	0	0	4	0	0	23	1	
Not available	1	7	7	3	0	0	1	51	4	
*(b)*										
*Resource allocation and outcomes in year 1 (first-year completers)*										
*n*	340	232	149	396	31	272	258	17	1695	
Overall treatment duration, days	538.3	521.8	642.2	652.2	736.0	527.8	570.4	530.9	578.5	<0.001
Ranibizumab injections	4.6	4.8	6.8	5.9	7.4	4.0	6.8	3.4	5.4	<0.001
Treatment switch, %	1	14	15	1	6	10	3	41	6	0.632
Ophthalmoscopies	3.3	7.6	6.0	8.7	0.6	1.9	1.6	1.8	4.8	<0.001
Optical coherence tomography	5.7	2.9	2.8	9.3	6.2	3.3	4.1	2.5	5.2	<0.001
Fluorescein angiography	1.2	1.3	0.9	0.5	1.9	2.1	0.2	0.6	1.0	<0.001
ICGA	0.4	0.1	0	0.1	0	1.1	0	0	0.3	<0.001
Monitoring visits^c^	3.9	3.9	1.3	4.3	1.7	5.3	2.4	4.9	3.7	<0.001
Clinic visits^c^	4.9	5.1	7.7	6.2	8.1	4.2	7.1	3.8	5.8	<0.001
Vision gainer, %^d^	17	17	28	30	11	13	31	25	23	<0.001
Vision maintained, %^d^	65	66	76	77	71	61	71	58	70	0.001
*Resource allocation and outcomes over 2 years (second-year completers)*										
*N*	240	136	107	350	18	159	163	11	1184	
Overall treatment duration, days	620.1	628.1	729.0	692.1	855.6	651.9	672.5	635.7	667.4	<0.001
Ranibizumab injections	7.4	7.2	12.1	9.5	15.6	6.2	10.9	4.2	8.9	<0.001
Treatment switch, %	2	15	13	1	11	11	4	64	6	0.749
Ophthalmoscopies	6.1	12.4	11.6	15.6	0.8	3.8	2.8	3.3	9.2	<0.001
Optical coherence tomography	10.9	6.3	6.1	17.5	12.1	6.8	7.9	5.2	10.9	<0.001
Fluorescein angiography	1.9	2.2	1.1	0.6	3.2	3.7	0.2	1.1	1.5	<0.001
ICGA	0.7	0.2	0	0.1	0	2.2	0	0	0.5	<0.001
Monitoring visits^c^	7.7	7.4	3.1	9.1	2.3	10.1	4.8	10.4	7.5	<0.001
Clinic visits^c^	8.1	8.1	14.6	10.2	17.3	6.7	11.7	6.1	9.8	<0.001
Vision gainer, %^d^	21	21	31	28	20	14	33	38	25	0.004
Vision maintained, %^d^	64	57	62	76	67	54	75	50	67	<0.001

Abbreviations: ICGA, indocyanine green angiography; VAS, visual acuity score.

Mean unless stated.

a *P*-value was derived from ANOVA test for continuous variables and *χ*^2^ test for categorical variables.

b Individual: reimbursement granted individually for this patient and treatment occasion; national standards: reimbursement according to national standards; independently: reimbursement granted for patient independently of how often treatment was provided.

c Monitoring (diagnostic assessments only) and clinic (scheduled treatment) visits. As this was an observational study, the actual number of treatments may differ to visits that were scheduled as clinic (treatment) visits, and the use of diagnostic tests, such as ophthalmoscopies, may have also occurred during clinic or monitoring visits, accounting for differences in numbers.

d Vision gainer (defined as patients who gained ⩾15 letters) and vision maintained (defined as no decline in visual acuity from baseline).

**Table 2:** Correlation between number of ranibizumab injections and candidate variables (all countries; over 2 years)

*Parameters*	*Coefficient*^a^	*SE*	*Lower 95% CI*	*Upper 95% CI*
Age at start of therapy	–0.0289	0.1141	–0.0378	0.056
Sex	0.0171	0.384	–0.0126	0.5193
Health insurance (public)	–0.028	0.1544	–0.053	0.0071
Reimbursement type (national standards)	0.1297	<0.001	0.1524	<0.001
Medical history (prior disease)	–0.0268	0.1711	–0.0354	0.0703
Medical history (concomitant disease)	–0.0003	0.9866	0.018	0.3595
Medical history (ocular disease)	–0.0246	0.2083	–0.0331	0.0905
Baseline VAS	–0.0088	0.6758	0.0424	0.0444
Treatment duration	**0.5645**	**<0.001**	**0.7389**	**<0.001**
Switch to other treatments	0.0275	0.1599	0.0632	0.0012
Baseline presence of retinal breaks^b^	NA	NA	NA	NA
Baseline presence of pigment epithelial detachment	0.0821	0.0038	0.1163	<0.001
Number of ophthalmoscopies	**0.2499**	**<0.001**	**0.3425**	**<0.001**
Optical coherence tomography, *n*	**0.3777**	**<0.001**	**0.4955**	**<0.001**
Fluorescein angiography, *n*	0.0509	0.0093	0.1154	<0.001
ICGA, *n*	–0.0341	0.0814	0.0302	0.1228
Ophthalmoscopies/optical coherence tomography, *n*	**0.3608**	**<0.001**	**0.4673**	**<0.001**
Number of monitoring visits	–0.1349	<0.001	0.0239	0.2224

Abbreviations: ICGA, indocyanine green angiography; NA, not available; VAS, visual acuity score.

a Pearson's correlation coefficient at 95% CI.

b No observation with retinal break episode. The bold values highlights the candidate instrumental variables.

**Table 3:** Relationship between number of ranibizumab injections and letter gains in year 1 (a) and over 2 years (b) tested by regression analysis using optical coherence tomography and ophthalmoscopy as an instrumental variable (all countries)

	*Coefficient*	*SE*	*Lower 95% CI*	*Upper 95% CI*	P*-value*^a^
*(a)*					
Number of injections (ranibizumab)	5.409	1.514	2.441	8.377	<0.001
Age at start of therapy	–0.106	0.081	–0.265	0.052	0.188
VAS at baseline	–0.279	0.037	–0.352	–0.206	<0.001
Constant	–3.760	14.380	-–31.945	24.426	0.794

*(b)*	*Coefficient*	*SE*	*Lower 95% CI*	*Upper 95% CI*	P*-value*^b^
Number of injections (ranibizumab)	1.933	0.552	0.852	3.015	<0.001
Age at start of therapy	–0.177	0.081	–0.335	–0.018	0.029
VAS at baseline	–0.391	0.035	–0.459	–0.322	<0.001
Constant	19.953	10.461	–0.550	40.455	0.056

Abbreviation: VAS, visual acuity score.

a Two-stage least-squares test: number of observations of the analysis was 1342, F-value: 16.57 (first-stage analysis), *R*^2^=0.0358.

b Two-stage least-squares test: number of observations of the analysis was 980, F-value: 19.68 (first-stage analysis), *R*^2^=0.057.

## References

[bib1] Ablonczy Z, Dahrouj M, Marneros AG. Progressive dysfunction of the retinal pigment epithelium and retina due to increased VEGF-A levels. FASEB J 2014; 28: 2369–2379. 24558195 10.1096/fj.13-248021 PMC3986839

[bib2] Brown DM, Kaiser PK, Michels M, Soubrane G, Heier JS, Kim RY et al. Ranibizumab versus verteporfin for neovascular age-related macular degeneration. N Engl J Med 2006; 355: 1432–1444. 17021319 10.1056/NEJMoa062655

[bib3] Rosenfeld PJ, Brown DM, Heier JS, Boyer DS, Kaiser PK, Chung CY et al. Ranibizumab for neovascular age-related macular degeneration. N Engl J Med 2006; 355: 1419–1431. 17021318 10.1056/NEJMoa054481

[bib4] Hariprasad SM, Morse LS, Shapiro H, Wong P, Tuomi L. Fixed monthly versus less frequent ranibizumab dosing and predictors of visual response in exudative age-related macular degeneration. J Ophthalmol 2012; 2012: 690641. 23251787 10.1155/2012/690641 PMC3515919

[bib5] Regillo CD, Brown DM, Abraham P, Yue H, Ianchulev T, Schneider S et al. Randomized, double-masked, sham-controlled trial of ranibizumab for neovascular age-related macular degeneration: PIER study year 1. Am J Ophthalmol 2008; 145: 239–248. 18222192 10.1016/j.ajo.2007.10.004

[bib6] Schmidt-Erfurth U, Eldem B, Guymer R, Korobelnik JF, Schlingemann RO, Axer-Siegel R et al. Efficacy and safety of monthly versus quarterly ranibizumab treatment in neovascular age-related macular degeneration: the EXCITE study. Ophthalmology 2011; 118: 831–839. 21146229 10.1016/j.ophtha.2010.09.004

[bib7] Holz FG, Amoaku W, Donate J, Guymer RH, Kellner U, Schlingemann RO et al. Safety and efficacy of a flexible dosing regimen of ranibizumab in neovascular age-related macular degeneration: the SUSTAIN study. Ophthalmology 2011; 118: 663–671. 21459217 10.1016/j.ophtha.2010.12.019

[bib8] Busbee BG, Ho AC, Brown DM, Heier JS, Suner IJ, Li Z et al. Twelve-month efficacy and safety of 0.5 mg or 2.0 mg ranibizumab in patients with subfoveal neovascular age-related macular degeneration. Ophthalmology 2013; 120: 1046–1056. 23352196 10.1016/j.ophtha.2012.10.014

[bib9] Martin DF, Maguire MG, Fine SL, Ying GS, Jaffe GJ, Grunwald JE et al. Ranibizumab and bevacizumab for treatment of neovascular age-related macular degeneration: two-year results. Ophthalmology 2012; 119: 1388–1398. 22555112 10.1016/j.ophtha.2012.03.053 PMC3389193

[bib10] Berg K, Pedersen TR, Sandvik L, Bragadottir R. Comparison of ranibizumab and bevacizumab for neovascular age-related macular degeneration according to LUCAS treat-and-extend protocol. Ophthalmology 2015; 122: 146–152. 25227499 10.1016/j.ophtha.2014.07.041

[bib11] Holz FG, Tadayoni R, Beatty S, Berger A, Cereda MG, Cortez R et al. Multi-country real-life experience of anti-vascular endothelial growth factor therapy for wet age-related macular degeneration. Br J Ophthalmol 2015; 99: 220–226. 25193672 10.1136/bjophthalmol-2014-305327 PMC4316940

[bib12] Holz FG, Tadayoni R, Beatty S, Berger A, Cereda MG, Hykin P et al. Key drivers of visual acuity gains in neovascular age-related macular degeneration in real life: findings from the AURA study. Br J Ophthalmol 2016, e-pub ahead of print 30 March 2016; doi:10.1136/bjophthalmol-2015-308166. 10.1136/bjophthalmol-2015-308166 PMC5256408 27030279

[bib13] Rakic JM, Leys A, Brié H, Denhaerynck K, Pacheco C, Vancayzeele S et al. Real-world variability in ranibizumab treatment and associated clinical, quality of life, and safety outcomes over 24 months in patients with neovascular age-related macular degeneration: the HELIOS study. Clin Ophthalmol 2013; 7: 1849–1858. 24092964 10.2147/OPTH.S49385 PMC3788820

[bib14] Brookhart MA, Rassen JA, Schneeweiss S. Instrumental variable methods in comparative safety and effectiveness research. Pharmacoepidemiol Drug Saf 2010; 19: 537–554. 20354968 10.1002/pds.1908 PMC2886161

[bib15] Greenland S. An introduction to instrumental variables for epidemiologists. Int J Epidemiol 2000; 29: 1102. 11101554 10.1093/oxfordjournals.ije.a019909

[bib16] Little RJ, Rubin DB. Causal effects in clinical and epidemiological studies via potential outcomes: concepts and analytical approaches. Annu Rev Public Health 2000; 21: 121–145. 10884949 10.1146/annurev.publhealth.21.1.121

[bib17] Martens EP, Pestman WR, de BA, Belitser SV, Klungel OH. Instrumental variables: application and limitations. Epidemiology 2006; 17: 260–267. 16617274 10.1097/01.ede.0000215160.88317.cb

[bib18] Staiger D, Stock JH. Instrumental variables regression with weak instruments. Econometrica 1997; 65: 557–586.

[bib19] Theil H. Principles of Econometrics. Wiley: New York, 1971.

[bib20] Zohoori N, Savitz DA. Econometric approaches to epidemiologic data: relating endogeneity and unobserved heterogeneity to confounding. Ann Epidemiol 1997; 7: 251–257. 9177107 10.1016/s1047-2797(97)00023-9

[bib21] Lin WY, Lee CC, Hsu CW, Huang KY, Lyu SR. Patients with knee osteoarthritis undergoing total knee arthroplasty have a lower risk of subsequent severe cardiovascular events: propensity score and instrumental variable analysis. PLoS One 2015; 10: e0127454. 26010912 10.1371/journal.pone.0127454 PMC4444196

[bib22] Maeda JL, Henke RM, Marder WD, Karaca Z, Friedman BS, Wong HS. Association between the unemployment rate and inpatient cost per discharge by payer in the United States, 2005-2010. BMC Health Serv Res 2014; 14: 378. 25311258 10.1186/1472-6963-14-378 PMC4283092

[bib23] Scientific Department The Royal College of Ophthalmologists. Age-related macular degeneration: guidelines for management. The Royal College of Ophthalmologists website http://www.rcophth.ac.uk/wp-content/uploads/2014/12/2013-SCI-318-RCOphth-AMD-Guidelines-Sept-2013-FINAL-2.pdf (accessed 26 April 2016).

[bib24] Schmidt-Erfurth U, Chong V, Loewenstein A, Larsen M, Souied E, Schlingemann R et al. Guidelines for the management of neovascular age-related macular degeneration by the European Society of Retina Specialists (EURETINA). Br J Ophthalmol 2014; 98: 1144–1167. 25136079 10.1136/bjophthalmol-2014-305702 PMC4145443

[bib25] Hassenstein A, Spital G, Scholz F, Henschel A, Richard G, Pauleikhoff D. [Optical coherence tomography for macula diagnostics. Review of methods and standardized application concentrating on diagnostic and therapy control of age-related macula degeneration]. Ophthalmologe 2009; 106: 116–126. 19156426 10.1007/s00347-008-1901-1

[bib26] Burgess S, Thompson SG. Improving bias and coverage in instrumental variable analysis with weak instruments for continuous and binary outcomes. Stat Med 2012; 31: 1582–1600. 22374818 10.1002/sim.4498

