# Supplementary material for: Determinants of visual acuity outcomes in eyes with neovascular AMD treated with anti-VEGF agents: an instrumental variable analysis of the AURA study
Source: Eye (Lond). 2016 May 20;30(8):1063–71. doi: 10.1038/eye.2016.90 (PMC4985685; doi:10.1038/eye.2016.90)

**SUPPLEMENT**

**Supplement Figure 1** Schematic that uses the number of OCT and ophthalmoscopy examinations performed as an instrumental variable to test the association between number of injections and effectiveness.

OCT, optical coherence tomography.


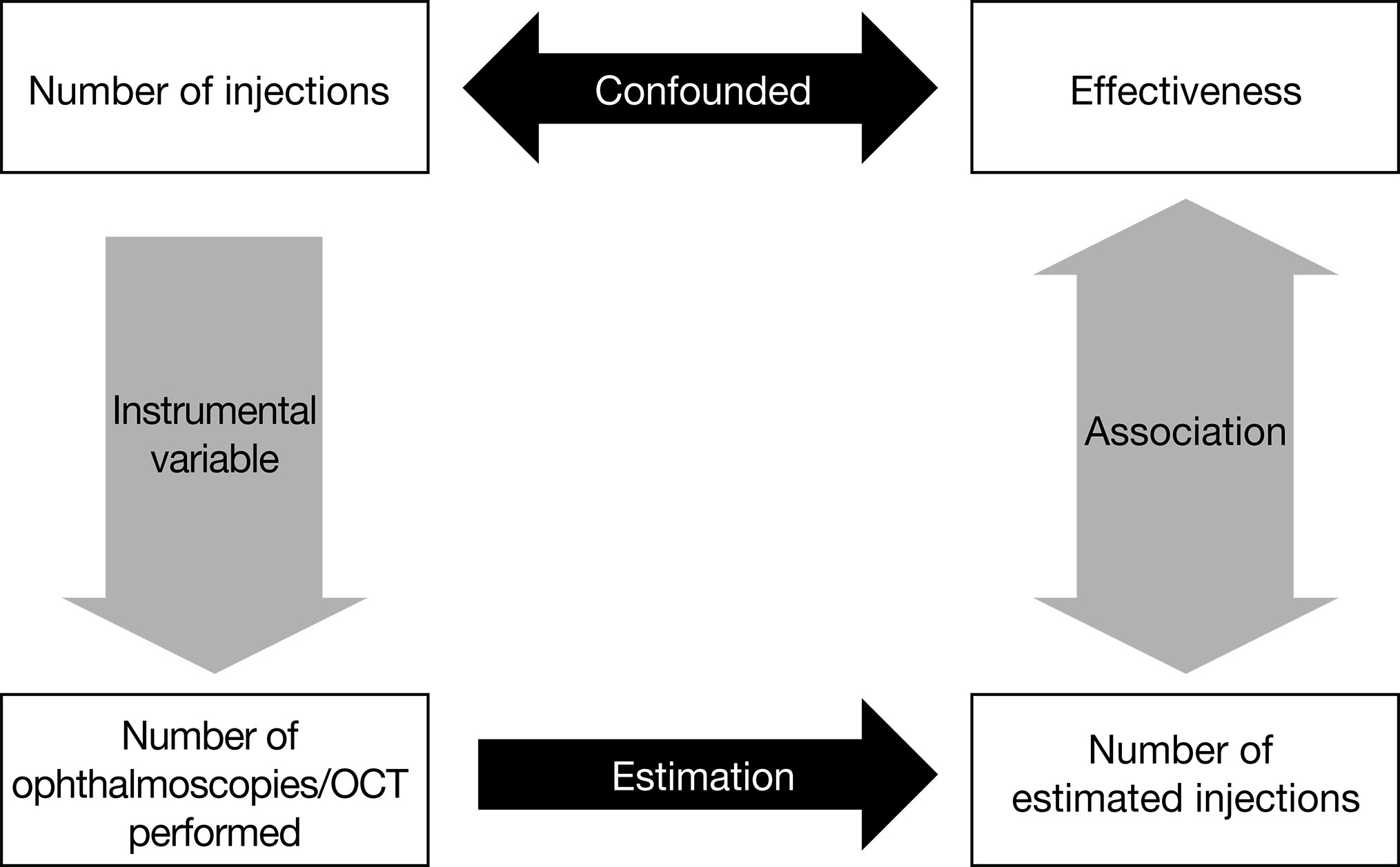

Supplement: Supplementary Information [file eye201690x1.docx]
